# Very High Refractive Index Transition Metal Dichalcogenide Photonic Conformal Coatings by Conversion of ALD Metal Oxides

**DOI:** 10.1038/s41598-019-39115-3

**Published:** 2019-02-26

**Authors:** Christopher T. Chen, Jacopo Pedrini, E. Ashley Gaulding, Christoph Kastl, Giuseppe Calafiore, Scott Dhuey, Tevye R. Kuykendall, Stefano Cabrini, Francesca M. Toma, Shaul Aloni, Adam M. Schwartzberg

**Affiliations:** 1 The Molecular Foundry, Lawrence Berkeley National Laboratory, 1 Cyclotron Road, Berkeley, California 94720 USA; 2 Università degli Studi di Milano-Bicocca, via R. Cozzi 55, 20125 Milano, Italy; 3 Joint Center for Artificial Photosynthesis and Chemical Sciences Division, Lawrence Berkeley National Laboratory, 1 Cyclotron Road, Berkeley, California 94720 USA

## Abstract

Materials for nanophotonic devices ideally combine ease of deposition, very high refractive index, and facile pattern formation through lithographic templating and/or etching. In this work, we present a scalable method for producing high refractive index WS_2_ layers by chemical conversion of WO_3_ synthesized via atomic layer deposition (ALD). These conformal nanocrystalline thin films demonstrate a surprisingly high index of refraction (n > 3.9), and structural fidelity compatible with lithographically defined features down to ~10 nm. Although this process yields highly polycrystalline films, the optical constants are in agreement with those reported for single crystal bulk WS_2_. Subsequently, we demonstrate three photonic structures - first, a two-dimensional hole array made possible by patterning and etching an ALD WO_3_ thin film before conversion, second, an analogue of the 2D hole array first patterned into fused silica before conformal coating and conversion, and third, a three-dimensional inverse opal photonic crystal made by conformal coating of a self-assembled polystyrene bead template. These results can be trivially extended to other transition metal dichalcogenides, thus opening new opportunities for photonic devices based on high refractive index materials.

## Introduction

Photonic devices rely on materials having high refractive index contrast between the photonic structure and the surrounding medium. The ease of deposition and reliable fabrication of a majority of the high refractive index materials (n > 3) using standard micro- and nano-scale lithographic techniques remains a challenge, particularly as feature sizes are reduced below 100 nm where surface roughness becomes a dominant factor. Higher refractive index materials have larger scattering cross sections^1^ and smaller mode volumes^2^, thereby constituting excellent candidates for light trapping elements in solar cells^3^ and photonic crystals^4^. The two dominant high index materials for photonic applications are crystalline silicon^5,6^ and gallium phosphide^7^. Silicon has the advantage of high refractive index from the visible well into the infrared, while GaP offers a similarly high index with the added advantage of low absorption well into the visible due to its large indirect bandgap. However, ideal material properties are difficult to achieve for the full range of two- and three-dimensional photonic structures. Two-dimensional photonic structures have the advantage of being easily integrated as an additive layer to optoelectronic devices or chip based photonics. Si-based two-dimensional photonic structures are readily fabricated from silicon on insulator substrates, and single crystal GaP requires epitaxial growth and layer removal/transfer before fabrication, limiting widespread application^8,9^. Three dimensional photonic crystals, due to the unique opportunities enabled by a complete photonic band gap, are also of great interest^5,10^. These structures are typically fabricated by conformally coating template structures prepared via self assembly^11^ or advanced lithography techniques^12^. Chemical vapor deposition of Si and GaP onto high surface area structures of various template materials (polystyrene beads, oxides) poses significant challenges to simultaneously achieving high material quality, conformal coverage, and uniformity^13^.

Layered transition metal dichalcogenides (TMDs) have returned to the research spotlight due to an intense focus on their remarkable optoelectronic properties when isolated as a single layer^14^. Importantly, these materials, in particular semiconducting phases of WS_2_^15^ and MoS_2_^16^, have long been known to have extremely high refractive indexes from measurements of bulk single crystals, higher than Si and GaP from the visible out to the infrared. In addition, our group has recently demonstrated that reliable synthesis of this class of materials is possible through careful control of humidity via a two-step process^17^. Specifically, this synthetic methodology comprises ALD of an oxide layer (e.g. WO_3_ and MoO_3_), and its conversion at high temperature in the presence of a H_2_S flow. Importantly, the presence of an oxide precursor layer enables reliable and precise lithographic patterning, which can be leveraged to produce complex structured templates which can be subsequently converted into very high index photonic structures. Despite their highly polycrystalline nature, thin films realized by this method have a refractive index similar to earlier measurements of bulk single crystals, exceeding Si and GaP. Three photonic structures are successfully demonstrated - first, a two-dimensional (2D) hole array made possible by patterning and etching an ALD WO_3_ thin film before conversion, second, an analogue of the 2D hole array first patterned into fused silica before conformal coating and conversion, and third, a three-dimensional inverse opal photonic crystal made by conformal coating of a self-assembled polystyrene bead template. Finally, the optical response of the 2D conformal structures are reproduced in full field optical simulations using the experimentally measured complex refractive index. These results support the further development and implementation of photonic structures based on transition metal dichalcogenides.

## Results

### Physical properties of WS2 thin films

**Figure 1:**
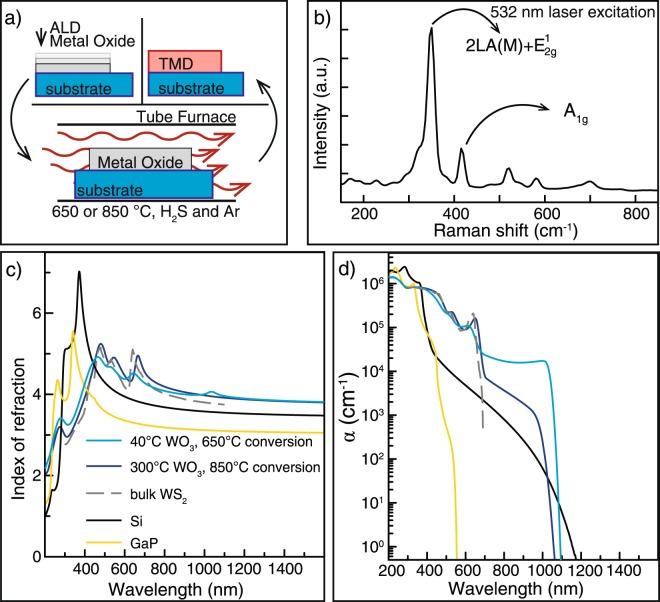
Synthesis, optical properties, and comparison of refractive indices of converted WS_2_ thin films. (**a**) Thin films of ALD metal oxides are heated in a chalcogen-containing gas environment at high temperatures to yield TMD thin films. (**b**) Representative thin film Raman spectrum. (**c**) Refractive index values of WS_2_ converted at 650 °C and 850 °C compared to literature values for bulk WS_2_^15^, Si^21^, and GaP (Woollam, J.A. GaP tabulated from UNL. *Unpublished*, *CompleteEASE Materials Library*.). Extracted refractive index values for thin film have features from the expected excitonic contributions and n > 4 for much of the visible into the IR. (**d**) Comparison of thin film absorption coefficient to literature values for bulk 3R-WS_2_, Si, and GaP. The absorption coefficient falls off near the expected indirect gap value of ~1.2 eV for both conversion temperatures.

Figure 1 provides an overview of the general chemical conversion process, a characteristic Raman spectrum, and a comparison of the refractive indices of converted WS_2_ thin films and literature values. As shown schematically in Fig. 1a, WO_3_ is readily converted into WS_2_ at high temperatures in the presence of elemental sulfur or H_2_S, as demonstrated widely in the literature^18,19^. To evaluate the optical properties of these films, WO_3_ thin films of about 30 nm thickness were deposited onto fused silica and thermal oxide coated silicon wafers with ALD. After annealing in H_2_S at high temperature, characteristic Raman modes in the Raman spectrum confirmed conversion into WS_2_ (Fig. 1b). Complex refractive indices were extracted by fitting spectroscopic ellipsometry measurements, with a focus on reproducing the expected excitonic and band edge features for WS_2_^20^. As shown in Fig. 1c, the extracted refractive indices for a film converted at 850 °C show strong excitonic contributions centered at 1.9 eV (653 nm) and 2.3 eV (539 nm) which we assign to the A and B exciton respectively. The absorption coefficient also appears to tail off close to the expected bulk WS_2_ bandgap value of 1.2 eV. The parameters of the fitted oscillator model are presented in Tables 1 and 2 to allow for full reproduction of the optical constants, and assignment of the individual contributions to the oscillator model is also presented in Table 1. The RMS surface roughness of thin films were measured to be 1–1.8 nm versus 0.6–0.8 nm as compared to the unconverted oxide by atomic force microscopy. Refractive indices and absorption coefficients of films converted at 650 °C and 850 °C are compared to literature values for WS_2_^15^, Si^21^, and GaP in Fig. 1c,d respectively.

**Table 1:** Oscillator parameters for ellipsometric model of 300 °C WO_3_ converted at 850 °C for 1 hour.

Assignment	Oscillator	Eo [eV]	Eg [eV]	Amplitude [a.u.]	Broadening [eV]	WR [eV] (right end point)	PR [eV] (right control point)	AR [a.u.] (right control point amp)	O2R [a.u.] (coeff)
Indirect bandgap	PSemi-M0	1.258	—	0.053	0.033	4.871	0.023	0.011	1.000
A exciton	Tauc-Lorentz	1.881	1.751	148.014	0.110	—	—	—	—
B exciton	Tauc-Lorentz	2.327	1.213	8.456	0.259	—	—	—	—
C exciton + direct gap	PSemi-M0	2.548	—	19.295	0.127	3.242	0.756	0.830	0.261
Higher order transition	PSemi-M0	4.369	—	5.205	0.260	10.224	0.801	1.614	1.000

See Methods for description of PSemi-M0 oscillator fit parameters.

**Table 2:** Other parameters for ellipsometric model of 300 °C WO_3_ converted at 850 °C for 1 hour.

Parameter	Value
𝜖∞	1.716
UV Pole Amplitude	76.458
UV Pole Energy	15.000 eV
IR Pole Amplitude	−0.007

**Figure 2:**
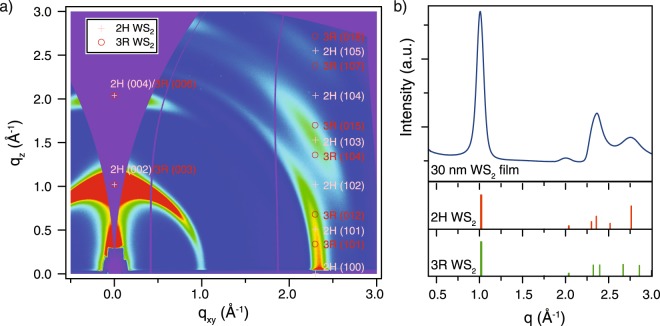
Structural characterization of WS_2_ thin film with grazing incidence wide angle X-ray scattering (GIWAXS). (**a**) GIWAXS pattern with superimposed peak locations for 2H and 3R-WS_2_. (**b**) Intensity spectrum derived by circular integration of GIWAXS pattern. Expected powder diffraction intensities plotted for 2H and 3R-WS_2_ below.

The structural properties of these films were studied with grazing incidence wide angle X-ray scattering (GIWAXS). Figure 2 depicts representative results from a thin film converted at 850 °C compared to expected diffraction patterns from bulk 2H and 3R-WS_2_^22^. As opposed to the metallic 1T phase, both the 3R and 2H polytypes are semiconducting, differing only by their respective stacking of individual van der Waals bonded layers, which in turn results in nearly identical optical properties^15,23,24^. In Fig. 2a, the GIWAXS pattern is overlaid with the expected peak positions from both structural polytypes. A fully polycrystalline film would yield diffraction rings that intersect each of these peak positions. As can be clearly seen for the (002) and (004) out-of-plane features, the signal intensity is concentrated in the q_z_ out-of-plane direction. This is a clear indication of a strong preferential texture, with a majority of the domains in the measurement area lying with their (002) planes flat to the substrate. Looking at the peaks with in-plane information, *i.e.* offset in q_xy_, the observed streaks can be clearly indexed as belonging to 2H-WS_2_. The scattering pattern was also integrated azimuthally to yield an intensity spectrum analogous to a typical diffraction spectrum (Fig. 2b). The kinematically predicted diffraction spectrum is plotted below for both structural polytypes. Note that the relative intensity of the peaks in the measurement will be different from the kinematic prediction due to the preferential texture. A visual inspection matches the intensity spectrum to the 2H structural polytype. A simple Scherrer analysis^25^ was applied by fitting Gaussians to the intensity spectrum, extracting domain sizes from 1.4–3.7 nm.

### Two-dimensional photonic crystals by etching and conformal deposition

**Figure 3:**
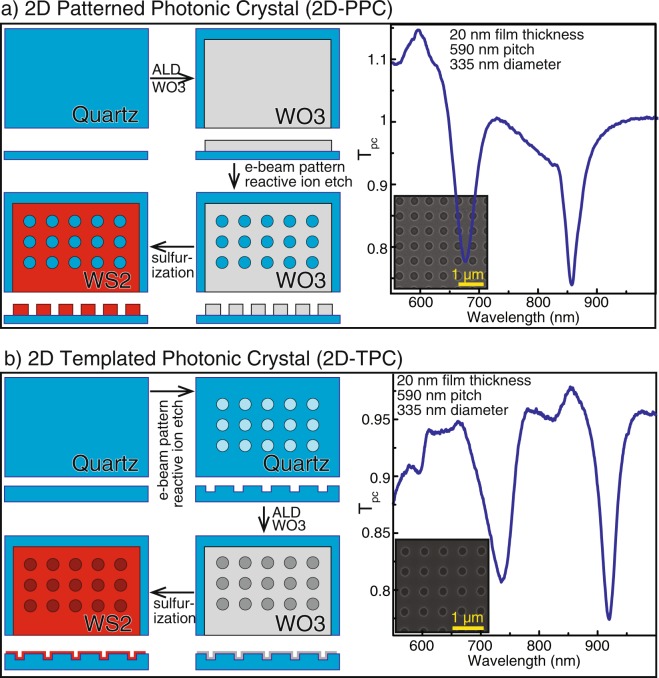
2D photonic structure fabrication and optical response. (**a**) Simple 2D patterned photonic crystal slabs can be prepared by patterning of the metal oxide followed by subsequent conversion. The normalized transmission at normal incidence (T_PC_) shows two strong features. (**b**) WS_2_ conformal coatings on quartz photonic crystal slabs yield similar T_PC_ phenomena.

A major advantage of our approach is in the facile fabricability of WO_3_ in contrast to direct etching of chalcogenides^26^ or other compound semiconductor materials. Traditional lithography techniques can be leveraged to precisely define the structuring of the oxide before conversion. In the first case, which we call a two-dimensional patterned photonic crystal (2D-PPC), a hole array structure is realized by electron beam lithography and reactive ion etching (RIE) on a thin film of ALD WO_3_ followed by high temperature sulfidization (Fig. 3a). The 2D-PPC in Fig. 3a is comprised of 335 nm diameter holes in a square grid with 590 nm pitch in a 20 nm thick WS_2_ film. Importantly, the fidelity of these structures after conversion matches that of the lithographically defined pattern due to careful control over the parameters of the conversion process^17^. The optical transmission spectra (T_PC_) of the two-dimensional patterned photonic crystals exhibit characteristic resonances. We define T_PC_ as the transmission of a photonic structure normalized by the transmission of an unpatterned thin film of identical thickness, such that values above unity can be interpreted as an anti-reflection effect. As shown later in detail, the positions of the transmission resonances match the theoretically calculated spectrum, confirming the high fidelity of the conversion process and validating the refractive indices extracted from the model fit to the ellipsometry data. In the second example, our process is applied onto a pre-fabricated structure that by itself does not exhibit strong photonic behavior due to the low refractive index of the template material (Fig. 3b). A similar approach has been used to modify the behavior of photonic crystal slab waveguides by ALD of TiO_2_^27^. Pits were defined on a fused silica wafer by electron beam lithography and subsequent RIE using a similar geometry to the 2D-PPC case. After conformal oxide deposition and conversion, these two-dimensional templated photonic crystals (2D-TPCs) behave very similarly to the 2D-PPCs. The 2D-TPC in Fig. 3b is a series of 335 nm diameter, 100 nm deep pits on a square grid with 590 nm pitch coated with 20 nm of WS_2_. The WS_2_ films prepared using the conversion process are of very high index, and the similar behavior of both subtractive (hole array) and additive (conformal) fabrication is a testament to the flexibility and fabricatability of this approach.

**Figure 4:**
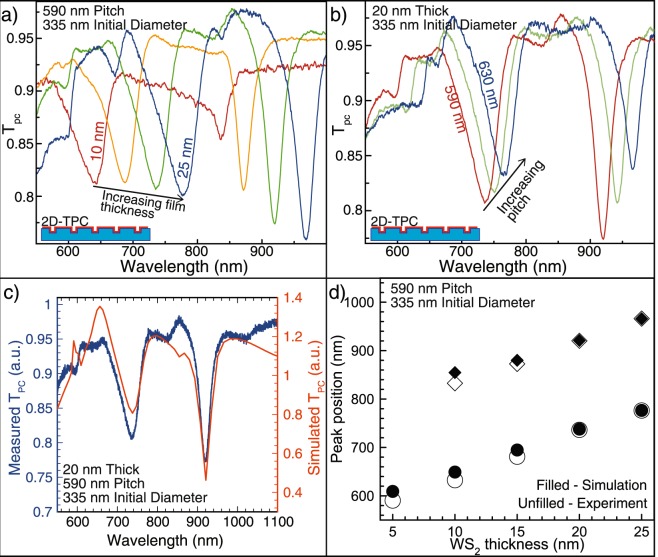
Further optical characterization of 2D-TPC structures and validation of measured optical constants with simulation. (**a**) Normalized transmission (T_PC_) spectra with increasing film thickness shows variation in observed features due to conformal modification of initial PC hole diameter. (**b**) T_PC_ also shifts as a function of the pitch of the initial structure. (**c**) Comparison of measured and simulated T_PC_ spectra for a given geometry shows excellent agreement in feature position. (**d**) Comparison of measured and simulated T_PC_ peak positions shows excellent agreement.

In Fig. 4, additional transmission measurements of 2D-TPCs of varying geometry are presented and compared to a full wave optical simulation of the identical structure. Due to the conformal nature of the TMD coating, increasing the thickness of the coating layer also modulates the diameter of the photonic structure. In Fig. 4a., the pitch and diameter of the arrays are held constant at 590 and 335 nm respectively, and a redshift of the features in the normalized transmission spectra is observed as the thickness of the layer is increased from 10 nm to 25 nm in 5 nm increments. In Fig. 4b, the film thickness and diameter are held constant at 20 nm and 335 nm respectively while the pitch spans 590, 610, and 630 nm, resulting in a smaller redshift in the normalized transmission spectra (Fig. 4b).

To validate the measured optical constants, we simulated the 2D-TPC structures with the finite difference time domain method as implemented in Lumerical FDTD Solutions. The refractive indices of the a thin film converted at 650 °C from 40 °C ALD WO_3_ (Fig. 1d) were used to best match the synthetic procedure used to prepare the 2D-TPCs, and a representative normalized spectrum for an array with 590 nm pitch, 335 nm diameter, and 20 nm film thickness is presented in Fig. 4c. The simulations show good agreement for the transmission feature dip positions across a wide range of thicknesses with template pitch and diameter held constant (Fig. 4d), but there are some discrepancies between the simulated and experimental spectra. Due to approximations in the refractive index model used by the simulation, the excitonic features are not perfectly reproduced, leading to some deviations in the shorter wavelength region. The exact values of the normalized transmission also differ from experiment, which is commonly seen in other broadband optical simulations. This result is a validation of the spectroscopic ellipsometry measurement and model fitting we performed to extract the WS_2_ optical constants and attests to the compatibility of the conformal coating and conversion technique with varying template geometry.

### Conformally coated three-dimensional inverse opal photonic crystal

**Figure 5:**
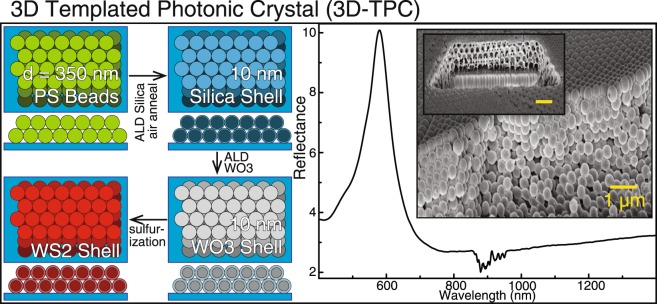
WS_2_ inverse opal photonic crystal. Self-assembled polystyrene beads are coated with SiO_2_ and WO_3_ and then converted into WS_2_ (left). Full infiltration of the 3D structure is achieved, as seen in the SEM micrographs (center), yielding a large spectral reflectance feature when measured in an integrating sphere (right). Inset scale bar measures 1 μm.

Finally, the conformal WS_2_ films were applied to a three-dimensional photonic crystal structure. As seen in Fig. 5, a self-assembled polystyrene bead (350 nm diameter) film with an opal structure is used as a template for deposition and conversion, leaving a 10 nm WS_2_/10 nm SiO_2_ inverse opal photonic crystal after completion of our process. Tilted SEM images of the structure and a FIB-milled cross section of the prepared structure show that despite the very large effective surface area of the template, the interior and exterior surfaces are coated, as is typical for ALD. After conversion, these structures have an intense spectral reflection feature arising from the stopband common to 3D photonic crystals^28^. This is the first demonstration of a WS_2_ 3D photonic crystal, enabled by the ability to prepare highly conformal WO_3_ coatings followed by transformation into very high index WS_2_. Application of this approach to other template designs^29,30^ should be easy to achieve.

## Discussion

The measured refractive index values in Fig. 1d are almost identical to those reported by Beal *et al*. in 1976 on bulk 3R-WS_2_ single crystals^15^, indeed exceeding the refractive index values for Si and GaP above ~425 nm. In contrast to recent reports which have focused on careful study of the optical properties of few-layer TMDs^24,31,32^ and prior measurements of bulk TMDs^15,16^, these thin films are polycrystalline with small domain size and thickness equivalent to many layers of material. The WS_2_ converted at 650 °C from an ALD thin film deposited at 40 °C has less intense excitonic response as compared to WS_2_ converted at 850 °C from an ALD thin film deposited at 300 °C. Achieving high refractive index WS_2_ despite low temperature ALD WO_3_ allows for the direct conformal coating of polymeric templates without disruption of template geometry, extending the possibilities for application to complex 3D photonic crystal structures^29,30^. There are no reports of the absorption coefficient for bulk WS_2_ at energies below the A exciton at ~2 eV down to the expected indirect bandgap at ~1.2 eV. It is important to note that this affects the comparison to the plotted literature values for 3R-WS_2_, as extraction of meaningful nonzero absorption coefficient values was not possible. In lieu of a direct comparison between our material and bulk WS_2_, it is clear that the high temperature conversion resulted in less absorption in the indirect gap dominated region from 650–1200 nm. This could be due to increased defect density due to the lower temperature conversion. Furthermore, while WS_2_ has an extremely high index, it is more absorbing in the visible and near-infrared as compared to both GaP and Si. Finally, it is important to note that there are important materials compatibility considerations to be made due to the sulfidization reaction done at high temperatures. For example, Si with a native oxide and ALD Al_2_O_3_ will undergo an unwanted chemical conversion into SiS_2_ and Al_2_S_3_, which adopt a filamentary structure and react with air to form H_2_S. These factors will have to be taken into account when designing a photonic structure based on this material process.

We have demonstrated fabrication of a scalable process for the conformal deposition of metal oxides and subsequent conversion into nanocrystalline TMD layers with very high refractive index. To illustrate possible photonics applications, three prototypical photonic structures were fabricated and characterized. While all of the examples presented are of WS_2_, this method should be generalizable to many of the oxide-chalcogenide combinations. For photonic applications that are sensitive to optical losses,* i.e.* parasitic absorption, other bulk TMDs and transition metal monochalcogenides with larger band gaps, such as ZrS_2_, SnS_2_^33^ and GaS, may offer significant advantages albeit at the cost of a lower refractive index compared to WS_2_. The scalability and fabricability of this process, combined with the possibility of extension to transition metal mono- and dichalcogenides, could open the door to a new class of photonic devices targeting the visible to near infrared.

## Experimental

### Material growth

Fused silica wafers were conformally coated with WO_3_ by plasma-enhanced ALD (PE-ALD, Oxford Instruments FlexAl) at *T* = 40 °C or 300 °C. The tungsten precursor (bis(tert-butylimido)-bis-(dimethylamido) tungsten)^34^ was dosed into the chamber for 3 seconds at 20 mTorr using 200 sccm argon as a bubbling carrier gas. The chamber was then isolated from the turbo pump and held at 30 mTorr for 1 second to compensate for the low reactivity of the precursor. The chamber and dosage line were then purged with 100 sccm of argon and pumped to 15 mTorr for 10 seconds. Oxygen plasma was generated via remote inductively coupled plasma (30 mTorr with 300 W power) and maintained for 2 seconds, then purged with argon at 15 mTorr for 10 seconds. This process was repeated as necessary to produce the required thickness. The growth rate for WO_3_ was ~0.6 Å per ALD cycle.

WO_3_ films were placed at the center of a 1-inch diameter single zone tube furnace. The furnace and samples were pumped/purged multiple times from low vacuum to atmospheric pressure under Ar flow (250 sccm 99.999% Ar, Praxair Ultra High Purity Grade). The temperature of the furnace was increased from room temperature to 650 °C or 850 °C over 10 minutes and kept at temperature for 60 minutes at atmospheric pressure. During heating, H_2_S (5 sccm 99.6% H_2_S, Praxair) was introduced at 300 °C and removed at the end of the 60 minute anneal. The furnace was then purged with Ar (250 sccm) during cooldown. After cooling to ~400 °C the furnace was opened for rapid cooling to ~120 °C over ~10 minutes.

### Characterization of the WS_2_ film

SEM micrographs were acquired with a Zeiss Ultra 60-SEM, detecting back-scattered electrons at 5 kV. Spectroscopic ellipsometry was performed with a J.A. Woollam M-2000. The wavelength-dependent complex refractive index was extracted from the ellipsometry data in the CompleteEASE software package by first fitting the TMD layer with a B-Spline and then parameterization with Tauc-Lorentz and parametric semiconductor^35^ (PSemi-M0) oscillators. The PSemi oscillators are Kramers-Kronig consistent and comprise four polynomial spline functions. PSemi-M0 contains seven fit parameters corresponding to amplitude (Amp), broadening (Br), width right (WR), control point position (PR), relative magnitude of the control point (AR), and the coefficient for the 2nd order terms in the polynomial (O2R). A complete description of these oscillators can be found in the J. A. Woollam WVASE software manual. Grazing incidence wide angle X-ray scattering (GIWAXS) was performed at beamline 7.3.3 of the Advanced Light Source with 10 keV photons at a grazing angle of 0.20° and a sample to detector distance of ~28 cm. Scattering patterns were collected using an area detector (Dectris Pilatus 1 M) with He sample chamber purging to minimize air scattering. Generation of calibrated scattering images and integrated spectra were performed using the Nika^36^ and WAXStools^37^ software packages. Fitting of the integrated spectra for Scherrer analysis was done in MagicPlot with Gaussian functions.

### Lithography and etching

Photonic crystal (PC) structures were patterned using e-beam lithography (Vistec VB-300) using ZEP 520 A (Zeon Corporation). The etching of the fabricated PC was performed with an Oxford Instruments Plasmalab80 Plus reactive ion etcher (RIE). WO_3_ was etched by a plasma consisting of 35 sccm CHF_3_ and 25 sccm Ar at 30 mTorr with 100 W power. SiO_2_ etching was carried out with a plasma composed of 96 sccm CHF_3_ and 4 sccm O_2_. The etching process alternated between 45 s at a power of 300 W and 45 s at a power of 25 W, repeating two times. The overall etching rate was ~0.5 nm s^−1^. The resist was stripped with Microchem Remover PG.

### 3D photonic crystal fabrication

Fabrication of large area, cm^2^-scale polystyrene (PS) opal templates over a wide diameter of PS spheres was performed using a previously published procedure^38^. In detail, 1 × 2 cm quartz substrates were first made hydrophilic by using the following cleaning procedure: (1) substrates were sonicated in a Alconox/DI water solution for 15 min, (2) then sonicated in pure DI water for 15 min, (3) next sonicated in a 1:1:1 acetone, ethanol, and DI water solution for 15 min. Finally, the substrates were treated with air plasma for ~5 min. Between each step, the substrates were inspected for dust/particulates which were removed via a combination of physical agitation with Kimwipes and flowing nitrogen, as necessary.

Non-functionalized polystyrene bead sizes of 350 nm (Polybead® Microspheres, 2.5% w/v in water) were purchased from Polysciences. PS bead solutions were diluted from their stock solutions to 0.05 v/v% with Millipore water. PS bead solutions were sonicated for 15 min before deposition.

The substrates were set tilted at a 60° angle (where 0° is horizontal) with each sample placed individually in small vials (1.5 cm diameter, 4.5 cm height). 1.5 mL of the PS bead solution was carefully dispensed into the vials by applying the pipette tip to the edge of the vial. The vial was placed on a hot plate and covered by a crystallization dish (15.0 cm diameter, 7.5 cm height) with a filter paper taped to the bottom so that droplets did not fall into the vials as water re-condensed on the glass. The hotplate was then set to 65 °C. The deposition was complete after 3 days. The substrates were then annealed on a hotplate at 90 °C for 10 min to remove any remaining solvent and ensure good contact of the beads with themselves and the substrate.

The opal templates were then coated with 10 nm of PE-ALD SiO_2_ at 40 °C. To remove the beads, an air anneal at 600 °C for 2 h was performed. Next, the SiO_2_ scaffold was coated with 10 nm of PE-ALD WO_3_. Finally, the composite WO_3_/SiO_2_ structure was annealed at 650 C for 1 h in H_2_S (5 sccm) and Ar (250 sccm) to yield a WS_2_/SiO_2_ inverse opal structure.

### Transmission and reflection measurements

Transmission measurements were performed on a home-built microscope. The light source (Thorlabs SLS201) was collimated and sent through the sample normal to the periodicity plane of the PCs. The transmitted light was then collected with a 40×, 0.75 NA, 0.66 mm working distance Nikon flat-field corrected fluorite objective and sent to an Andor Shamrock SR303i spectrometer coupled to an Andor Newton CCD camera. The total integrated collection area was approximately 100 × 100 µm^2^. Light transmitted by an unstructured, equally thick WS_2_ film on fused silica was used as a reference to which the PC transmission spectrum was normalized. In this way, the residual contributions to reflection and absorption of the WS_2_ are eliminated and any residual signal is due to photonic resonances. We define the ratio between the light transmitted by the PC (${I}_{{PC}}$) and that transmitted by the unstructured thin film (${I}_{0}$) to be the transmission of the PC (T_PC_).

Spectral reflectance measurements were performed on a Shimadzu SolidSpec-3700 UV/Vis/NIR spectrometer using an integrating sphere. The baseline for the reflectance measurement was collected with a silver mirror (ThorLabs PF10-03-P-01). All measurements used an aperture with a 6 mm spot size.

### Modeling

Full wave finite-difference time domain (FDTD) simulations of the 2D templated photonic crystal realized by conformal coating of the patterned fused silica wafer were implemented in the Lumerical FDTD Solutions software package. The complex refractive index of the fused silica and WS_2_ were derived from literature values and fitting of ellipsometry data as described above. Normal incidence broadband plane wave illumination was used with periodic boundaries in the lateral directions and absorbing boundaries in the vertical directions. The reflection and transmission of the structure were monitored using field power monitors placed above the illumination source and below the surface of the fused silica wafer.
